# Supplementary material for: The fate of Candida tropicalis in the black soldier fly larvae and its nutritional effect suggest indirect interactions
Source: PLoS One. 2025 Jul 3;20(7):e0325056. doi: 10.1371/journal.pone.0325056 (PMC12225849; doi:10.1371/journal.pone.0325056)
Supplement: Attachment — Submitted filename: response to reviewers.docx [file pone.0325056.s002.docx]

Dear anonymous reviewers, thank you for your comments, which considerably improved our manuscript. You can find below a point to point respond to the comments:

Reviewer 1

1. The title uses the term "non-orthodox symbiotic" without providing a clear definition in the article. It is a highly specific term that requires a precise explanation and data confirmation.

*We removed this term from the title and discussion and changed it to “indirect interactions” as the effect of the C. tropicalis on the BSF seems to be through the substrate*

2. There is inconsistent use of professional terminology throughout the article, such as fungi, yeast, and C. tropicalis. And the terminology for black soldier fly. The article uses "black soldier fly," "BSF," and "Hermetia illucens." Although these are all correct, the full name and abbreviation should be given at first mention, and then one term should be used consistently.

*We agree, we improved the term’s consistency in the manuscript by refereeing to C. tropicalis and BSF*

3. This article is based on the study by Kannan et al. (2023). The authors used different C. tropicalis feeding strategies for BSF larvae. However, was there a significant increase in BSF body weight after supplementing their diet with C. tropicalis? Could the authors provide supporting evidence for this effect?

*Unfortunately, we did not measure the larval body mass in this experiment*

4. Line 214: The meaning of significant differences between A and B was not mentioned in the article

*The meaning of significance was added to the figure legends*

5. Line 209-215: There is a lack of a unit for the y-axis in Figure 2.

*The amount of DNA was compared to a relative sample, therefore it is not quantification but relative measurement.*

6. How many BSF were used in Figure 2a and Figure 2b.
The article doesn't clearly state the sample size and number of replications for each experiment. Small sample sizes could affect the reliability and generalizability of results.

*The sample sizes were added throughout the manuscript*

7. How did the authors confirm that the separated gut sections were correctly identified as the anterior midgut, middle midgut, and posterior midgut? Did the authors have anatomical data or specific markers for AM, MM, and PM to validate these gut sections?

*The gut parts were separated according to the physiological description provided by Bonelli et al. (2019) and were ensured accurate identification of the midgut, using the pH levels known to maintained in each region (lines 128-131)*

8. Line 216-221: The methodology for Figure 3 was not described in the Materials and Methods section.

*The methodology explained in lines 93-104*

Please provide information about the source and purity of standards used for quantification.

*The relevant information was added*

The fatty acid abbreviations on the x-axis in Figure 3 lack detailed explanations. Please clarify whether the y-axis represents weight percentage or molar percentage of total fatty acids.

*The figure axis was changed accordingly*

9. Line 285-289: The conclusions drawn by Genta et al. (2009) appear to contradict the arguments presented by the authors in this paper. This discrepancy needs to be addressed and explained.

*If the lysozyme afect the fungal survival, we suggest that C. tropicalis can reduce the enzymatic activity of the insect to allow fungal survival. It is describes in lines 305-308*

10. Line 287: Please demonstrate the relationship between lysozyme activity and the survival rate of BSF larvae.

*We meant the survival of the fungi (corrected in the text)*

11. The authors should clarify whether C. tropicalis enhances or suppresses the immune response in BSF larvae.

*The effect of Candida on the insect immune system is specific in line 307*

Additionally, several genes associated with BSF survival have been previously identified. Could you examine and discuss the potential relationship between these known survival-related genes and the immune response observed in the discussion?

*It is not clear to us which genes that related to the survival of the BSF the reviewer suggests. The effect of the effect of fungi on the immune response in discussed in the paper by Herman et al. 2024.*

Reviewer 2

1. Nutritional composition of BSFL Is changeable and can be regulated using different feed stuff. A little discussion on this aspect could improve the Introduction section. For example: [doi.org/10.3920/JIFF2021.0162](http://doi.org/10.3920/JIFF2021.0162), 10.1093/ee/nvab135

*These references were added and the topic is discussed in the introduction (lines 57-59)*

2. Details for equipment and software used in the experiment should be unified: the name of manufacturer and its location should be given.

Add separate subsection in section 2 with chemicals and materials. Provide information on chemicals purity and manufacturers.

*The information was added (not in a separate section but in the text)*

3. Tables can be modified. It is better to add separate columns with data on investigated values for experimental and control groups of BSF larvae.

*As the information of the LCMS contains a vast number of metabolites, usually the analysis is conducted as a comparison between the treatments and the control. Therefore the tables present only the significant difference in these comparisons*

4. As suggestion, all figures can be combined to the one figure. In this regard, it will be visually comfortable for readers to compare the main results obtained.

*As the figures present different analyses of different treatments we think it will be more distracting to combine them.*

5. The text should be carefully checked for typos and grammatical errors.

*The text was checked thoroughly*
