# Supplementary material for: The fate of Candida tropicalis in the black soldier fly larvae and its nutritional effect suggest indirect interactions
Source: PLoS One. 2025 Jul 3;20(7):e0325056. doi: 10.1371/journal.pone.0325056 (PMC12225849; doi:10.1371/journal.pone.0325056)
Supplement: Attachment — Submitted filename: Review Comments.docx [file pone.0325056.s003.docx]

6. Review Comments to the Author

Please use the space provided to explain your answers to the questions above. You may also include additional comments for the author, including concerns about dual publication, research ethics, or publication ethics. (Please upload your review as an attachment if it exceeds 20,000 characters)

Reviewer #1: This manuscript provides an interesting and valuable study on the relationship between the fungus C. tropicalis and BSF. The work makes a meaningful contribution to understanding insect-microbe interactions in an economically important insect species. However, there are several points that could be improved before publication:

Methodology should be clear
1. The experimental design is somewhat complex with different treatments. While Fig 1, the text could more clearly explain the rationale behind the 10-day with C. tropicalis followed by 1-day without design. What was the hypothesis behind this specific timing?

An additional explanation was added to the method section (lines 114-116)

2. The authors use the 26S rRNA gene to assess the presence of C. tropicalis in the gut, but this approach may require further clarification:
1) Authors should consider using absolute quantitative PCR to establish standard curves for more accurate quantification of fungal amounts.
2) Additional methods, such as culture counts or microscopic observation, may be needed to validate the results.
3) Therefore, providing more details on how C. tropicalis was quantified would strengthen the methods section.

We conducted relative quantification and the method was better described (lines 139-141). Unfortunately, we did not conduct any additional methods to support the results.

3. The manuscript would benefit address more information about how data normality was assessed before choosing between parametric and non-parametric tests.

The relevant data was included (lines 199-201)

The evidence is insufficient to support what this study demonstrates.
4. The paper suggests C. tropicalis manipulates BSF digestive enzyme production, but the evidence is primarily correlative. Authors should either provide stronger evidence for this claim or acknowledge its speculative nature more explicitly.

The paragraph was changed accordingly (lines 296-309)

5. The research data in this study is insufficient to support conclusions. The limited dataset does not provide adequate evidence to make such definitive claims about the relationship between C. tropicalis and BSF larvae. More comprehensive investigations would be needed to establish these mechanisms with certainty.

A disclaimer was added in the discussion (lines 322-323)

Reviewer #2: The authors considered all comments and recommendations. The revised manuscript deserves further consideration by Editorial board.
